# Building Research Capacity in Low- and Middle-Income Countries and Pandemic Preparedness: Lessons Learned and Future Directions

**DOI:** 10.4269/ajtmh.23-0675

**Published:** 2024-01-23

**Authors:** Peter H. Kilmarx, Karen A. Goraleski, Erum Khan, John F. Lindo, Nancy Gore Saravia

**Affiliations:** ^1^ Fogarty International Center, U.S. National Institutes of Health, Bethesda, Maryland;; ^2^ American Society of Tropical Medicine and Hygiene, Arlington, Virginia;; ^3^ Department of Pathology and Laboratory Medicine, Aga Khan University, Karachi, Pakistan;; ^4^ Department of Microbiology, University of the West Indies, Kingston, Jamaica;; ^5^ Centro Internacional de Entrenamiento e Investigaciones Medicas (CIDEIM), Cali, Colombia;; ^6^ Universidad Icesi, Cali, Colombia

## Abstract

Research capacity is a critical component of pandemic preparedness, as highlighted by the challenges faced during the Ebola outbreak in West Africa. Recent global initiatives, such as the Research & Development Task Force of the Global Health Security Agenda and the World Health Assembly’s resolution on strengthening clinical trials, emphasize the need for robust research capabilities. This Perspective discusses the experiences of leaders in infectious disease research and capacity building in low- and middle-income countries, focusing on Colombia, Jamaica, and Pakistan. These case studies underscore the importance of collaborative efforts, interdisciplinary training, and global partnerships in pandemic response. The experiences highlight the necessity for rapid pathogen identification, capacity for genomic sequencing, and proactive engagement with policymakers. Challenges faced, including the shortage of trained staff and reliance on imported reagents, emphasize the ongoing need for building research capacity.

## INTRODUCTION

Research capacity is a critical element of pandemic preparedness. This was clearly articulated in a report titled *Money and Microbes*, which was sponsored by the World Bank and the Coalition for Epidemic Preparedness Innovations after the challenges experienced during development of medical countermeasures for Ebola in 2014–2016 due to relatively weak research infrastructures in the highly affected West African countries.^1^ In 2021, the Global Health Security Agenda established a Research & Development Task Force to create metrics that can be used as part of a joint external evaluation to assess national health research capacity.^2^ And in 2022, the World Health Assembly adopted a resolution (WHA75.8) on “strengthening clinical trials to provide high-quality evidence on health interventions and to improve research quality and coordination.”^3^ As noted in these documents, with sufficient research capacity scientists can focus on national health research priorities, including infectious and noncommunicable diseases, and then pivot in case of a new health threat, as we saw with COVID-19.

In contrast to the challenges with lack of research capacity when Ebola began spreading in Guinea, there was a relatively strong research response in Brazil when the Zika outbreak was identified in 2015; substantial progress was made in rapidly characterizing the epidemiology and newly recognized, diverse clinical manifestations. Another counterexample was the research response to the COVID-19 pandemic, which included countries with strong research capacity and led to the rapid development of medical countermeasures, although the research was not well coordinated and was quite inefficient. Unfortunately, however, the risk of a weak research response and delayed development of medical countermeasures in the event of a health emergency remains in many parts of the world. To date, there is not a consensus set of metrics or a definition of the minimum or ideal research capacity countries should have to meet national needs or for preparedness. This is the focus of ongoing work by the Global Health Security Agenda and the WHO.^2^^,^^3^

In this Perspective, leaders in infectious disease research and research capacity building share their experiences in establishing research capacity in low- and middle-income countries and describe how that capacity was deployed during the COVID-19 pandemic. The authors are engaged in diverse research areas, including neglected tropical diseases, viral diseases, and microbiology, and represent very diverse settings from Asia to Latin America and the Caribbean.

### Colombia.

Vector-borne infections, particularly the arboviruses dengue, chikungunya, and Zika, as well as malaria and cutaneous leishmaniasis have been a source of epidemic outbreaks in Colombia during the past two decades. In March 2020, COVID-19 was first detected in Colombia; by April 2022, reported cases had surpassed 6 million and more than 135,000 deaths had occurred.^4^ Genomic surveillance supported by a collaborative multi-institution network established by the Colombian National Institutes of Health allowed the detection of multiple introductions from other countries and the conduct of phylodynamic analyses.

Colombia is the site of the Fogarty International Center Global Infectious Diseases (GID) research training program developed by the Centro Internacional de Entrenamiento e Investigaciones Medicas (CIDEIM) in partnership with the Yale School of Public Health on leishmaniasis and other vector-borne and emerging infectious diseases. Ecological and epidemiological settings in Colombia, located at the intersection of Central and South America, include the presence of diverse, globally important transmissible diseases. The CIDEIM originated in 1961 as a bilateral Technical Assistance Mission with Tulane University and is supported by the U.S. NIH’s International Center for Medical Research and Training program. National commitment was instrumental in the continuity of the program, which was subsequently established in 1990 as a nonprofit autonomous Colombian institution dedicated to infectious disease research and training.^5^ The CIDEIM is now located within the campus of Universidad Icesi and is strategically allied with this institution and its associated medical center, Fundación Valle del Lili.

The GID research training program initially focused on strengthening basic research capacity in immunology, molecular biology, and genomics applied to leishmaniasis and other vector-borne infections, followed by strengthening translational and implementation research. National support for institutional development together with international and national research funding was crucial in building a state-of-the-art infrastructure with biosafety level (BSL)-3 facilities and a multidisciplinary research team that includes social scientists and communicators who facilitate engagement with communities, health authorities, and other stakeholders in addressing health priorities.

Substantial research capacity has been achieved, shared, and strengthened through inter-institutional cooperation and leveraging of existing resources. In addition to career development through mentored research, the articulation of GID research training with postgraduate programs in national and regional universities has been strategic in establishing sustainable research capacity. Research-focused courses offered as electives within established postgraduate curricula promote “peer-to-peer” learning among investigators, faculty, and students in diverse institutions using an inter-institutional, web-based format. This approach to capacity building, together with short courses/workshops designed to institutionalize transversal skills applicable across the spectrum of research and sectors (e.g., effective project planning and evaluation, good health research practices, principles of implementation research),^6^ enabled integration of the CIDEIM into the multi-institutional response to COVID-19.^7^ The prior participation of personnel from national and local public health institutions in these capacity-building activities, both as trainees and faculty, fostered intersectoral cooperation and networking.

The CIDEIM partnered with the Colombian National Institutes of Health in designing and conducting the national SARS-COV-2 seroprevalence survey and served as a member of the national COVID-19 diagnostic network.^8^ Technologies implemented for research on vector-borne diseases through prior training in institutions with established facilities and experience in genomic and bioinformatics analyses were crucial to the participation of CIDEIM in local and national responses for the diagnosis of COVID-19 and genomic surveillance of emergent variants. COVID-19 demanded concerted responses across sectors; translational and implementation research capacity provided platforms for intersectoral initiatives for diagnostics and therapeutics.^9^^,^^10^ Approaches to building research capacity that have articulated existing competencies, infrastructures, knowledge, and technologies and investigators who developed their careers within this context have laid the scientific, technical, and collaborative foundation for CIDEIM to effectively respond to the COVID-19 pandemic and to future health challenges.

### Jamaica.

The Caribbean has been plagued with several epidemics of viral diseases including dengue in 2012, Chikungunya in 2014, and dengue and Zika in 2015. These were followed by the SARS-CoV-2 pandemic in 2020, which by 2023 resulted in more than 150,000 reported cases and approximately 3,500 deaths. These epidemics overlaid epidemics of HIV and chronic noncommunicable diseases. The University of the West Indies (UWI) in Jamaica has played a key role in viral surveillance, as it houses the National Influenza Center, which provides weekly reports to the Surveillance Unit of the Ministry of Health. Jamaica lacked the human capacity in virology to adequate respond to these epidemics, as there were too few trained virologists dedicated to research in the country. One mechanism to build this capacity was the establishment of the State University of New York (SUNY) – University of the West Indies (UWI) Health Research Consortium. This dual–university systems initiative to develop research capacity was established by agreement between SUNY, UWI, and the Ministry of Health and Wellness, Jamaica.^11^

The SUNY-led team obtained a 5-year Fogarty GID with a sub-award to UWI to train 10 Jamaican scientists at the master’s and doctoral degree levels in virology research. This GID program was recently renewed for 5 years with one of the authors at UWI (J. F. L.) as a principal investigator. The areas of focus include viral discovery in both humans and mosquito vectors, chronic viral infection, sexually transmitted infections (excluding HIV), and immunometabolism.^12^ Training includes an overlay of the current degree research of these candidates, such as specialized laboratory attachments in the United States, responsible conduct of research, and grant writing. The overall aim of the program is to produce the next generation of virologists who can compete successfully for extramural funding while building independent research careers. The program has been supplemented by collaboration with other entities, including the Global Virus Network, the Abbott Pandemic Defense Coalition, and Rush University.

The COVID-19 pandemic disrupted travel to the United States for several of the scholars in the program, and training was restricted to Jamaica and supported by Zoom. Building on the capacity established through the training program, several SARS-CoV-2 research projects were conducted, including areas of diagnosis and vaccination^13^^,^^14^ and a study of long COVID in the pediatric population. An important outcome of the research activity in virology at UWI was the decision of the Ministry of Health and Wellness to support the establishment of a Next-Generation Sequencing Service at the Department of Microbiology to track the COVID-19 pandemic. Prior to the setting up of the service, samples were shipped to Caribbean Public Health Laboratories in Trinidad and Tobago for sequencing. With the next-generation sequencing service at UWI, results could be provided on a weekly basis to inform public health activities.

### Pakistan.

In Pakistan, perennial major infectious disease threats have included dengue, typhoid fever, hepatitis B and C, and tuberculosis. Aga Khan University (AKU), established in 1983, was Pakistan’s first private, not-for-profit university. Over the past decade, Aga Khan University has invested considerably in research capacity building as part of its mission to develop human capacities through the discovery and dissemination of knowledge and its application through service. The organization includes highly secure BSL-2 and BSL-3 laboratories equipped with a tissue culture room and whole genome sequencing facility as well as clinical trial and animal research units. As a lead university in Pakistan, AKU played an instrumental role in the response to the COVID-19 pandemic. The Department of Pathology and Laboratory Medicine (PALM) has had a long-standing track record of researchers working on cross-cutting research themes. With 47 faculty members representing a mix of clinical pathologists, clinical scientists, immunologists, and molecular biologists, the department was well positioned to leverage clinical and research laboratories for translation of evidence-based research into diagnostics.

Early in the pandemic, AKU faculty contacted international collaborators at Public Health England to establish a rapid molecular diagnostic assay for the novel coronavirus. By mid-February 2020, the clinical laboratory at AKU validated and launched SARS-CoV-2 polymerase chain reaction (PCR) testing, and the Pakistan National Institutes of Health contacted AKU to collaborate and report PCR testing results to the national data base.

International collaborators and research funding agencies, including the Fogarty International Center, Health Security Partners, the United World Antiviral Research Network of the Centers for Research in Emerging Infectious Diseases program, and WHO-Pakistan, helped establish the diagnostic assay through exchange of protocols and reagents. Efforts to optimize whole genome sequencing assays were prioritized, and by June 2020, AKU had sequenced the first SARS-CoV-2 genome.^15^

Abbott International also approached PALM-AKU to initiate a clinical trial to validate a diagnostic test, which would not have been possible in Pakistan without the previously established research infrastructure and active collaboration.

The AKU initiated several other COVID-19 response activities. With the Government of Sindh, Pakistan, PALM-AKU shouldered SARS-CoV-2 molecular testing and variant detection. The number of samples soared to a peak of 2,000 per day in June 2021. The AKU already had laboratorians trained in molecular techniques on the research side who were mobilized to the clinical laboratory to assist in its province-wide COVID-19 surveillance program. The need for rapid diagnostics to initiate field surveillance became evident from the outset of the pandemic. By August 2021, a number of commercial kits for rapid antigen and antibody testing became available on the market in Pakistan. The Government of Pakistan–Sindh Province sought AKU assistance in validating the accuracy of these kits. Most of these validation studies required use of archived pre–COVID-19 sample testing.

The AKU identified and reported SARS-CoV-2 variants through targeted PCR and genomic sequencing assays. This work was supported by AKU Special Provost Funds, Pakistan Higher Education Commission grants, and the WHO-Pakistan country office. The AKU has been the largest provider of SARS-CoV-2 genomes into global initiative on sharing all influenza data (GISAID) from Pakistan and provides regular reports to health authorities.^16^^,^^17^

Although the use of N-95 masks was mandated for clinical services, clinicians had not undergone prior fit testing. The AKU Biosafety Officer and Institutional Biosafety Committee performed fit testing for approximately 2,000 clinical faculty and staff members. In addition, the committee formulated an in-house solution to be used in fit testing in the context of global lockdowns and shipment delays.^18^ This safety measure gave a level of confidence to staff when attending patients, preventing cross-transmission of infection. Faculty members at AKU served as advisors to government task forces, including Sindh’s COVID-19 Research Committee and COVID-19 Testing Capacity Enhancement Committee and the Prime Minister’s Health Task Force.

Some of the challenges AKU faced during the COVID-19 pandemic were a lack of trained staff for specialized testing and reliance on imported reagents. The spread of false information on social media pertaining to COVID-19 was also a major challenge.

## CONCLUSION

These experiences underscore the pivotal role of research capacity in pandemic preparedness, offering valuable insights from Colombia, Jamaica, and Pakistan. These case studies emphasize the significance of collaborative efforts, interdisciplinary training, and global partnerships for effective crisis response. The lessons learned, such as the need for rapid pathogen identification, in-country capacity for genomic sequencing, and proactive engagement with policymakers, offer a road map for strengthening research infrastructure and enhancing readiness for future pandemics. When the research ecosystem pivots to address a health emergency, there is a downside in that there is slower progress on pre-emergency research objectives; however, this is similar for all systems that must pivot in an emergency. Ideally, in a crisis, the most critical activities are continued as overall objectives are reprioritized.

The imperative to build research capacity in low- and middle-income countries is well understood by the global research community. Additional efforts are needed to educate other stakeholders about the value of and progress on building this capacity. In today’s environment of hyper-attention to costs and return on investment, it may be beneficial to train researchers on key messages and how they can play a more active role in making the case for the value of the research effort in their immediate communities and more broadly. Without a deeper understanding of capacity building and its downstream effect on health security, researchers may be leaving valuable teaching moments on the cutting-room floor. As the world navigates ongoing health challenges, these insights serve as essential building blocks for a more resilient, coordinated, and successful global response.
